# Ambiguity of Residual Constraint-Based Precise Point Positioning with Partial Ambiguity Resolution under No Real-Time Network Corrections Using Real Global Positioning System (GPS) Data

**DOI:** 10.3390/s20113220

**Published:** 2020-06-05

**Authors:** Honglei Qin, Peng Liu, Li Cong, Xia Xue

**Affiliations:** School of Electronic and Information Engineering, Beihang University, No. 37 Xueyuan Road, Beijing 100191, China; ateqhl@buaa.edu.cn (H.Q.); blairliu@buaa.edu.cn (P.L.); xuexiaphd@buaa.edu.cn (X.X.)

**Keywords:** partial ambiguity resolution, precise point positioning, fractional cycle bias, network correction, convergence time

## Abstract

Although precise point positioning (PPP) is a well-established and promising technique with the use of precise satellite orbit and clock products, it costs a long convergence time to reach a centimeter-level positioning accuracy. The PPP with ambiguity resolution (PPP-AR) technique can improve convergence performance by resolving ambiguities after separating the fractional cycle bias (FCB). Now the FCB estimation is mainly realized by the regional or global operating reference station network. However, it does not work well in the areas where network resources are scarce. The contribution of this paper is to realize an ambiguity residual constraint-based PPP with partial ambiguity resolution (PPP-PARC) under no real-time network corrections to speed up the convergence, especially when the performance of the float solution is poor. More specifically, the update strategy of FCB estimation in a stand-alone receiver is proposed to realize the PPP-PAR. Thereafter, the solving process of FCB in a stand-alone receiver is summarized. Meanwhile, the influencing factors of the ambiguity success rate in the PPP-PAR without network corrections are analyzed. Meanwhile, the ambiguity residual constraint is added to adapt the particularity of the partial ambiguity-fixing without network corrections. Moreover, the positioning experiments with raw observation data at the Global Positioning System (GPS) globally distributed reference stations are conducted to determine the ambiguity residual threshold for post-processing and real-time scenarios. Finally, the positioning performance was verified by 22 GPS reference stations. The results show that convergence time is reduced by 15.8% and 26.4% in post-processing and real-time scenarios, respectively, when the float solution is unstable, compared with PPP using a float solution. However, if the float solution is stable, the PPP-PARC method has performance similar to the float solution. The method shows the significance of the PPP-PARC for future PPP applications in areas where network resource is deficient.

## 1. Introduction

As precise point positioning (PPP) was proposed by Zumberge et al. to realize positioning solution with only a stand-alone receiver, and it is used normally in double-frequency observations [1,2,3]. However, the convergence time is typically 30 min [2].

To reduce the convergence time in a single constellation Global Navigation Satellite System (GNSS), the simplest method is to reduce the level of pseudo-range noise by means of observation combinations theoretically [4,5,6,7,8]. However, different observation combinations have a similar performance which is presented by Liu and Qin [9,10,11].

Due to the fractional cycle biases (FCBs) in the Global Positioning System (GPS) observations are absorbed by the nondifferential ambiguity estimates, so their integer properties are destroyed [12,13]. FCB leads to greatly reducing the efficiency of PPP ambiguities searching, and makes it difficult for the filtering algorithm to converge in a short time. Moreover, FCB will also interfere with the filter update and brings in the wrong prior information.

The most fundamental method is ambiguity fixing. As early as 1999, Gabor and Nerem first proposed the algorithm for ambiguity-fixing in a stand-alone receiver [14]. At that time, due to the existence of selective availability (SA) and the impact of satellite orbits and clock error accuracy on the narrow-lane ambiguity evaluation, they did not realize the PPP with ambiguity resolution (PPP-AR). Gao and Shen attempted to realize ambiguity pseudo-fixing [4]. Subsequently, there are six PPP-AR methods [15] where three existing PPP-AR methods are Ionosphere-free [16,17,18]. They is a single difference between the Uncalibrated Phase Delay/Fractional Cycle Bias (UPD/FCB) model [14], decoupled satellite clock (DSC) model [12] and integer recovery clock (IRC) model [19]. Ge et al. partially improved Gabor’s algorithm and proposed an ambiguity-fixing method based on FCB on the premise of the short-term stability of FCB [13].

The narrow-lane FCB varies from 2 to 3 h [16]. In the case of the FCB estimation in a stand-alone receiver, the estimated error of FCB is relatively large, so that the ambiguities can not be fixed accurately [20]. Some researchers use observation models with the assistance of a continuously-operating reference station (CORS) [21]. It is usually used to realize PPP with real-time kinematic (PPP-RTK) [9,15,19,22,23,24,25,26,27,28,29]. The convergence time has been reduced to make it more practical.

PPP-AR needs real-time network corrections by the regional or global operating reference station network [9,28,30,31], compared with the float solution. When there is no network resource in special positioning scenarios, ambiguity-fixing becomes nearly impossible. In the post-processing PPP, Hu proposed that the narrow-lane FCB is updated once per 15 min, namely, narrow-lane FCB is calculated with 15-min segment data in a stand-alone receiver, and then the narrow-lane FCB is used to calculate the ambiguity fixed solution in this segment [32]. The positioning solution is better than PPP with floating ambiguity. In the Real-time PPP, the estimated error of FCB is relatively larger and FCB cannot be estimated as same as that in post-processing PPP. The real-time PPP-AR can not be realized under no real-time network corrections. In this scenario, the convergence time of any other methods will be longer. However, compared with the float solution, whether is there an algorithm to reduce the convergence time under no real-time network corrections, especially when the performance of the float solution is poor?

To solve the above problem, we propose an ambiguity residual constraint-based precise point positioning with partial ambiguity resolution (PPP-PARC) to improve the convergence speed under no real-time network corrections. Experimental verification is conducted by real GPS data. The primary contributions of the paper are summarized as follows.

1. An improved FCB update strategy is presented to satisfy requirements of real-time PPP without the assistance of real-time network corrections.
2. An ambiguity residual constraint-based precise point positioning with partial ambiguity resolution (PPP-PARC) is proposed to fix partial ambiguities successfully so that convergence time can be reduced under no real-time network corrections.
3. A PPP experiment is operated to analyze the performance of the new algorithm in post-processing and real-time PPP.

## 2. Partial Ambiguity Fixing

### 2.1. Non-Integer Ambiguity

The conventional ionosphere-free combining observation model is

(1) $$\begin{array}{cc} L_{IF} & =\;f_{1}^{2}/\left(f_{1}^{2}-f_{2}^{2}\right)L_{1}-f_{2}^{2}/\left(f_{1}^{2}-f_{2}^{2}\right)L_{2}\\ & =\;\rho +cd_{P_{IF}}^{r}-cd_{P_{IF}}^{s}+T_{W}-\lambda_{IF}N_{IF}+b_{L_{IF}}^{r}-b_{L_{IF}}^{s}-b_{P_{IF}}^{r}+b_{P_{IF}}^{s}+\varepsilon_{L_{IF}}, \end{array}$$

where $L_{IF}$, $L_{1}$, and $L_{2}$ are observations with different frequencies, $f_{1}$ and $f_{2}$ are the signal frequencies of observations. ρ is the distance between receiver and satellite. $dt_{P_{IF}}^{r}$ is receiver clock error. $dt_{P_{IF}}^{s}$ is satellite clock error. Tw is the projection of tropospheric zenith wet path delay. $N_{IF}$ is the ambiguity of carrier phase. $\lambda_{IF}$ is the wavelength of carrier phase. $b_{P_{IF}}^{s}$, $b_{P_{IF}}^{r}$, $b_{L_{IF}}^{s}$, and $b_{L_{IF}}^{r}$ are hardware delay of pseudorange and carrier phase in receiver and satellite.

The hardware delay with the uncalibrated phase is represented by Uncalibrated Hardware Delay (UHD) [12,13,16], where the integer part is represented by UPD and the fractional part is represented by FCB.

The UPDs can be absorbed into the ambiguities, there is no influence for both the integer characteristics of ambiguities and the positioning solution. However, When the FCBs are absorbed into the ambiguities, the integer characteristics of the ambiguities are destroyed.

To converge quickly, the most direct way is the ambiguity pseudo-fixing. It does not consider the carrier hardware delay, the ambiguities are fixed into integers coercively. However, there may be one cycle deviation between the estimated ambiguity and the real ambiguity. If the positioning accuracy is not required to be so high, the ambiguity pseudo-fixing may accelerate convergence and reach decimeter-level positioning.

Another method is ambiguity-fixing based on FCB.

(2) $$B_{}^{s}=N_{r}^{s}+b_{r}^{}-b_{}^{s}+\varepsilon_{n}.$$

After the single difference between satellites,

(3) $$B_{sd}^{s}=N_{sd}^{s}+b_{sd}^{s}+\varepsilon_{sdn}.$$

The variation of wide-lane UPD with a single difference between satellites is relatively stable for several months [13]. However, the variation of narrow-lane UPD with a single difference between satellites is not stable within a day, so the ambiguities can be fixed by dividing a day into several stable segments for narrow-lane UPD with a single difference between satellites.

### 2.2. Ambiguity Residual Constraint-Based PPP-PAR

**Figure 1:**
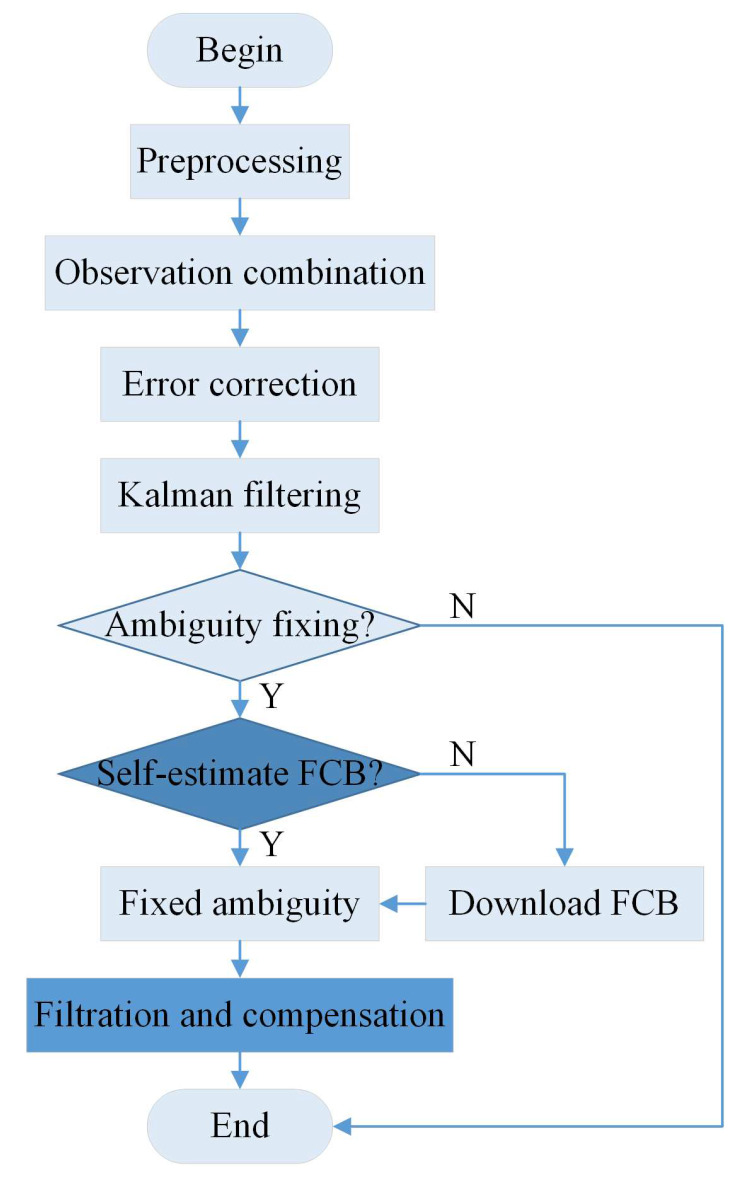
The flowchart of precise point positioning (PPP)-partial ambiguity resolution (PAR) without the assistance of the real-time network corrections.

The flow chart of Precise Point Positioning can be described as shown in Figure 1. The PPP with float resolution needs a long convergence time. In order to converge fast, adding the GNSS system is a feasible method, and another method is ambiguity resolution which does not need to add a data source. However, conventional ambiguity resolution needs FCB estimated by the network. When the communication network is poor, there is no assistance in real-time network corrections.

We tried to estimate FCB by a stand-alone receiver. Due to the FCB estimated by a stand-alone receiver being worse than that estimated by CORS network, not all ambiguities can be fixed, so partial ambiguity-fixing with strict constraints is more practical. Compared with conventional PPP-PAR, FCB estimated by a stand-alone receiver should have a different update period. Meanwhile, there are more strict constraints in screening fixed ambiguity.

## 3. FCB Estimation

### 3.1. System Architecture

**Figure 2:**
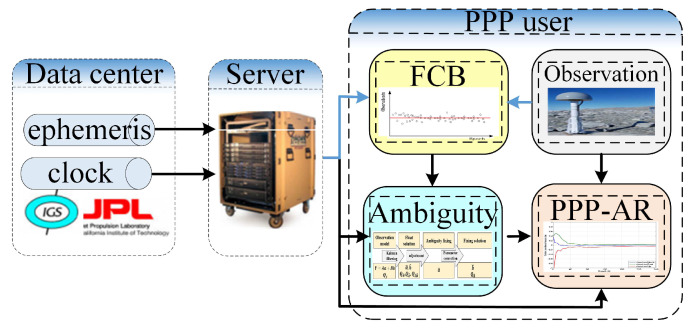
PPP positioning system without the assistance of the real-time network corrections.

In order to achieve ambiguity resolution without the assistance of real-time network corrections, the FCB self-estimation model should be added as shown in Figure 2. Compared with conventional fixed solution, the user system should estimate FCB itself first, and then the integer character of the ambiguities can be recovered, the ambiguities can be fixed, finally, PPP with ambiguity resolution is used to reduce convergence time. The FCB estimated by a stand-alone receiver is described in detail as follows.

### 3.2. FCB Estimation Algorithm

Due to receiver FCB was not taken into account, the FCB was not accurate enough. Therefore, the single-differential ambiguities between the satellites should be used to eliminate the influence of receiver FCB [33]. In other words, the single-differential FCB related to satellite FCB could be calculated according to the reference satellite FCB. Then, the single-differential FCB is used to realize the positioning solution with ambiguity fixing.

Due to the narrow-lane satellite FCB only being stable for 2 to 3 h, its estimation segment is normally set to 15 min (less than 30 min) [16]. Although the wide-lane satellite FCB is relatively stable and its estimation segment can be set to 24 h, wide-lane satellite FCBs vary with the reference satellite, so they are uniformly set to 15 min (same as the narrow-lane satellite FCBs). First, the fractional part of M-W combining ambiguity $B_{MW}$ is separated as the wide-lane FCB by GPHASE function where the fraction is subjected to the Von Mises distribution and its range is [−0.5, 0.5] [14]. According to the ionosphere-free combining float ambiguity $B_{IF}$, both the wide-lane float solution and narrow-lane float solution are obtained. Then the wide-lane and narrow-lane ambiguity can be fixed after the ambiguity satisfies the screening condition.

### 3.3. The Improved FCB Update Strategy

So far, single-differential FCBs estimated in a stand-alone receiver are only suitable for the post-processing PPP. If the single-differential FCBs can be used for the subsequent epoch, the real-time problem can be solved. There are two problems when single-differential FCBs are estimated in a stand-alone receiver. One is that the update period of single-differential FCBs is too long, and the other is that the accuracy of single-differential FCBs is worse.

If the FCBs update is 15 min, the ambiguities cannot converge in the first 15 min, so the first estimated FCB cannot be applied to the ambiguity-fixing PPP. The ambiguity fixed solution needs to adopt the second 15-min FCB which is estimated by a stand-alone receiver. Thus, it causes the fixed solution to possibly occur after the positioning lasts for 30 min. At that time, if the ambiguity float solution has already converged, the ambiguity-fixed solution makes no sense. On the contrary, if the ambiguity float solution has not converged, the convergence time of the fixed solution will be more than 30 min, so that the fast convergence character of the fixed solution does not work.

**Figure 3:**
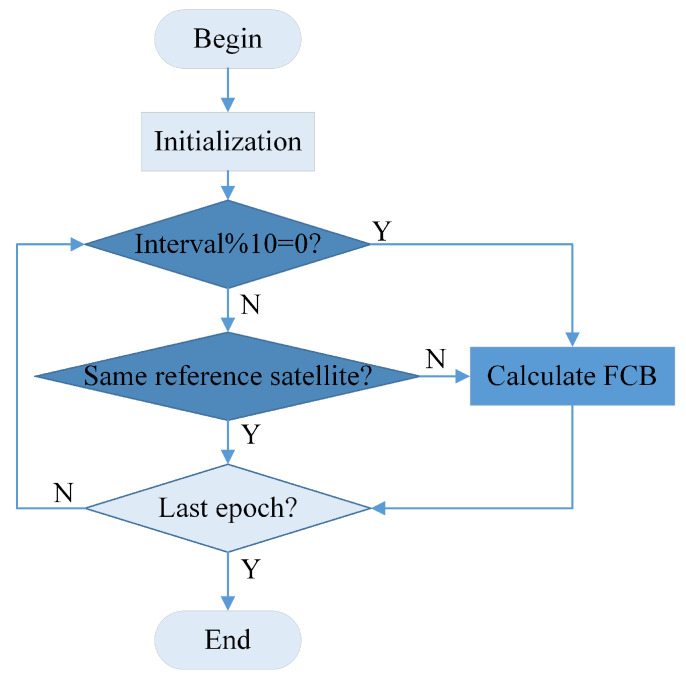
The flowchart of fractional cycle bias (FCB) estimation algorithm.

It is considered to reduce the update interval of single-differential FCB to 5 min (10 epochs). Then the single-differential FCB can be used to recover the integer ambiguities, and the ambiguity fixed solution can be calculated as shown in Figure 3. The single-differential FCB accuracy is worse so that the correctness of fixed ambiguities cannot be guaranteed, and further ambiguity screening is necessary as follows.

## 4. Partial Ambiguity Resolution

### 4.1. Ambiguity Residual Characteristic

As a stand-alone receiver is used to estimate FCB, the noise will be introduced in the FCB. It may have a negative impact on the correctness of FCB, resulting in poor performance of the ambiguity-fixing.

According to the equivalent observation equation theory which is proposed by Xu [34], the GNSS observation equation can be transformed into the one that only has the ambiguity parameters as follows.

(4) $$V_{fd}=L-\left[E-H_{1}{\left(H_{1}^{T}RH_{1}\right)}^{-1}H_{1}^{T}R\right]H_{2}X_{2,fd},$$

where $V_{fd}$ is the observation residual, *E* is unit matrix, *R* is the covariance matrix of observation noise, $X_{2,fd}$ is the estimated ambiguity parameters, $H_{2}$ is the corresponding design matrix, $H_{1}$ is the remaining part of design matrix *H*, which is the design matrix for all estimated parameters [10].

Provided that $D=\left[E-H_{1}{\left(H_{1}^{T}RH_{1}\right)}^{-1}H_{1}^{T}R\right]H_{2}$, then the Equation (4) can be expressed as follows.

(5) $$V_{fd}=L-DX_{2,fd}.$$

Provided that the ambiguity residual is

(6) $$V_{se}=X_{2,ft}-X_{2,fd}$$

The above Formula (5) can be transformed as

(7) $$\begin{array}{cc} V_{fd} & =L-D\left(X_{2,ft}-V_{se}\right)\\ \; & =L-DX_{2,ft}+DV_{se}\\ \; & =V_{ft}+DV_{se} \end{array}$$

The accuracy is related to ${V_{fd}}^{T}RV_{fd}$. So

(8) $$\begin{array}{cc} y= & {V_{fd}}^{T}RV_{fd}\\ = & {V_{ft}}^{T}RV_{ft}+{V_{se}}^{T}D^{T}RV_{ft}+{V_{ft}}^{T}RDV_{se}+{V_{se}}^{T}D^{T}RDV_{se} \end{array}$$

#### 4.1.1. Monotonicity

According to the Matrix partial derivative theory, it takes partial derivative of y with respect to $V_{se}$,

(9) $$\frac{\partial y}{\partial V_{se}}=2{v_{ft}}^{T}RD+2{V_{se}}^{T}D^{T}RD.$$

When the value of Equation (9) is 0, say $\frac{\partial y}{\partial V_{se}}=0$, $V_{se}$ can be obtained as follows.

(10) $$V_{se}=-{\left(D^{T}RD\right)}^{-1}D^{T}Rv_{ft}.$$

Here, it can be seen obviously that the variation of $V_{se}$ is closely related with that of $V_{ft}$. When the value of $V_{se}$ is close to the value of Equation (10), that is $V_{se}=-{\left(D^{T}RD\right)}^{-1}D^{T}Rv_{ft}$, the *y* has a minimum or maximum value, the positioning accuracy is highest or lowest.

#### 4.1.2. Convexity

We clarify the convexity of the Equation (8) as follows.

(11) $$\frac{\partial^{2}y}{\partial {V_{se}}^{2}}=2D^{T}RD.$$

According to the definition of quadric form in linear algebra, the matrix product form $D^{T}RD$ is assuredly the positive definite matrix in Equation (11). In other words, *y* is a convex function, and *y* will reach its minimum value when the value of Equation (9) is 0.

We can conclude that when the ambiguity residual $V_{se}$ is equal to the value of Equation (10), that is $V_{se}=-{\left(D^{T}RD\right)}^{-1}D^{T}Rv_{ft}$, or it can be said that the ambiguity residual $V_{se}$ is around $-{\left(D^{T}RD\right)}^{-1}D^{T}Rv_{ft}$, the quadratic sum of the fixed solutions residuals *y* in Equation (8) can reach its trough or minimum. Due to the distribution of $V_{ft}$ is the normal distribution about real float solution, when $V_{se}$ locates near the centralized value (real value of FCB), the *y* will reach its trough or minimum, the convergence time may be reduced.

### 4.2. Ambiguity Subset Screening

According to the ambiguity residual characteristic, the closer $V_{se}$ is to real value FCB, the better the positioning performance of PPP-AR is. Provided that

(12) $$V=V_{se}-b_{sd}^{s},$$

where $b_{sd}^{s}$ is the estimated FCB. *V* should be as small as possible. If the boundary of *V* is restricted during ambiguity subset screening, correctness of the selected ambiguities can be guaranteed.

(13) $$|V|<V_{th},$$

where $V_{th}$ is the limit threshold, and also $V_{th}>0$. According to GPHASE function, the range of threshold $V_{th}$ is [−0.5, 0.5]. The detail value of threshold $V_{th}$ should be obtained by controlled variable experiment.

**Figure 4:**
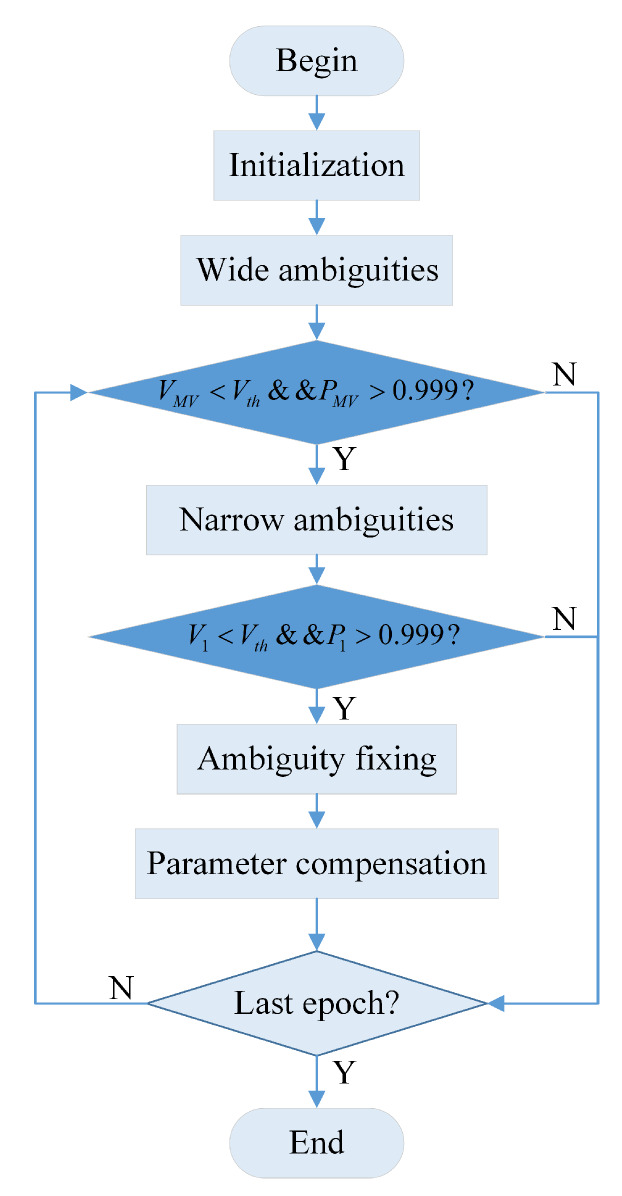
The flow chart of ambiguity fixing algorithm.

Based on the conventional ambiguity success rate constraint in screening the ambiguity subset [35,36], the ambiguity fixing algorithm adds the threshold $V_{th}$ constraint as shown in Figure 4. $V_{MW}$, $V_{1}$ are the wide-lane ambiguity residual and narrow-lane ambiguity residual in the current epoch, respectively. $V_{th}$ is the ambiguity residual threshold constraint. $P_{MW}$, $P_{1}$ are the wide-lane ambiguity success rate and narrow-lane ambiguity success rate in the current epoch, respectively.

## 5. PPP Experiment

### 5.1. Experimental Configuration

The positioning experiment was conducted by using the self-developed software based on the prototype RTKLIB2.4.3. All error corrections are shown in Table 1.

The ratio of the pseudorange to the carrier phase was 100 [37]. Meanwhile, the initial values of X,Y,Z and dT and their corresponding variance–covariance were the least-squares solutions of the first epoch. In the first epoch, the Hopfield model was used to calculate the initial value of Tw, and the corresponding variance–covariance was set as any value (0.25). Ambiguities $N_{1},N_{2}$ and ionosphere $I_{1}$ were derived from the non-difference observation equation, and the corresponding variance–covariance followed the covariance propagation rule [4]. The dynamic noise variance of receiver clock error was 900 m${}^{2}$/s${}^{2}$, the dynamic noise variance of tropospheric zenith wet delay was 10${}^{-8}$ m${}^{2}$/s${}^{2}$, ionospheric dynamic noise variance was $10^{-6}$ m${}^{2}$/s${}^{2}$ [10].

(14) $$\begin{array}{cc} I_{1}=\; & f_{2}^{2}/\left(f_{1}^{2}-f_{2}^{2}\right)\left(P_{2}-P_{1}\right)\\ \lambda_{1}N_{1}= & P_{1}-L_{1}-2I_{1}=P_{1}-L_{1}-2f_{2}^{2}/\left(f_{1}^{2}-f_{2}^{2}\right)(P_{2}-P_{1} \end{array}$$

(15) $$\begin{array}{cc} =\; & \left(1+2f_{2}^{2}/\left(f_{1}^{2}-f_{2}^{2}\right)\right)P_{1}-2f_{2}^{2}/\left(f_{1}^{2}-f_{2}^{2}\right)P_{2}-L_{1} \end{array}$$

(16) $$\begin{array}{cc} \lambda_{2}N_{2}= & 2f_{1}^{2}/\left(f_{1}^{2}-f_{2}^{2}\right)P_{1}+\left(1-2f_{1}^{2}/\left(f_{1}^{2}-f_{2}^{2}\right)\right)P_{2}-L_{2}. \end{array}$$

The adopted data files and parameter configurations are shown in Table 2, where ocean tide was not corrected because the 11 tidal wave parameters were missing. The time interval of precise satellite orbit and clock product was 15 min and 30 s, respectively. The post-processing PPP used final ephemeris igs, and the real-time PPP used ultra-rapid ephemeris igu. To verify convergence performance, we only intercepted the first two hours of observation data in the Renix files for the experiment.

### 5.2. Post-Processing PPP Experiment

#### 5.2.1. Stations Selection

To verify the availability of the ambiguity residual constraint-based PPP-PAR method (PPP-PARC), we first chose the stations which have two things in common. One is that PPP with float resolution is unstable, especially the up direction, because the up direction has worse accuracy than both east and north directions. The up error determines the convergence time. The other is that the weather is cloudy. The former makes it possible to optimize the float solution. The latter guarantees that observations are not so bad that ambiguities cannot be fixed. If the method does not optimize float solution, in this case, the method could be invalid in all cases.

**Figure 5:**
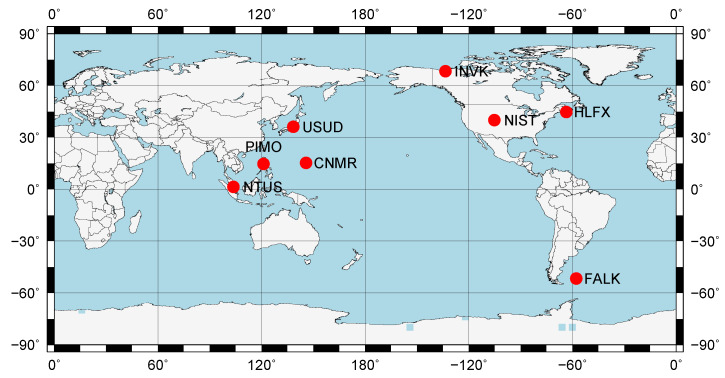
Station distribution in the post-processing PPP.

There are 499 globally-distributed reference stations at the Crustal Dynamics Data Information System (CDDIS), of which 358 stations have the week solution of coordinate in the file igs18P1996_all.ssc. According to the two constraints, we chose the stations around pan-pacific (CNMR, FALK, HLFX, INVK, NIST, NTUS, PIMO, and USUD) shown in Figure 5.

#### 5.2.2. Determination of the Threshold

**Figure 6:**
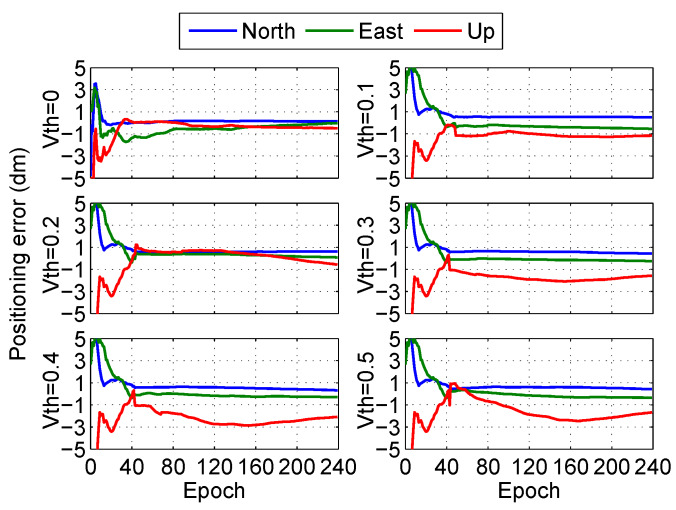
Positioning error of fixed solution in different thresholds at station CNMR in the post-processing PPP.

Taking the CNMR station as an example, the influences of the different thresholds $V_{th}$ on the PPP-PARC are analyzed with a step length of 0.1 cycles as shown in Figure 6, where the threshold $V_{th}=0$ is a float solution and the threshold $V_{th}=0.5$ is a pseudo fixed solution.

**Figure 7:**
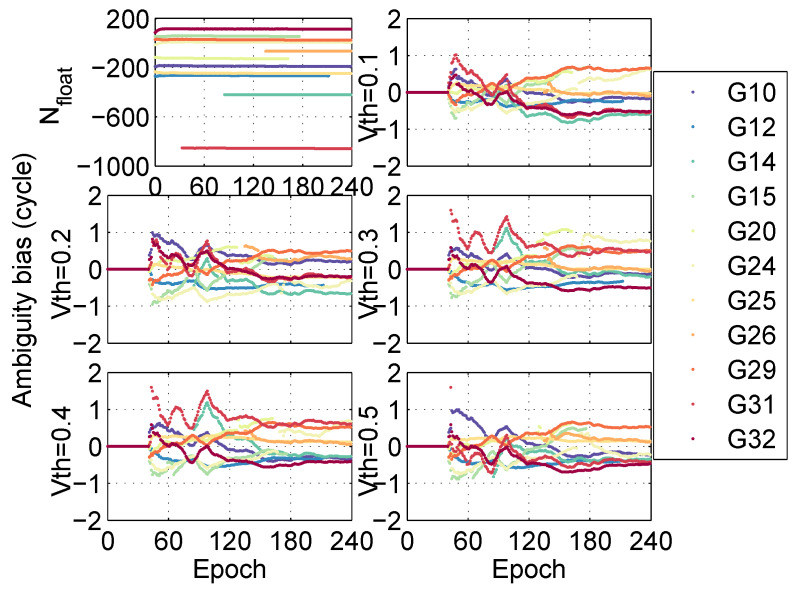
Ambiguity bias in different thresholds at station CNMR in the post-processing PPP.

According to the convergence time from Figure 6, it can be seen that the positioning performance is the best when the threshold $V_{th}=0.2$. Meanwhile, the residuals between fixed ambiguities and floating ambiguities under different thresholds are analyzed, as shown in Figure 7. It is found that when the threshold $V_{th}=0.1$ or 0.2, the residuals between fixed ambiguities and floating ambiguities are all within 1 cycle, but Root Mean Square (RMS) of ambiguity resolution in $V_{th}=0.2$ is the best. The conclusion is consistent with the positioning performance. In the post-processing PPP, we set the threshold $V_{th}=0.2$.

#### 5.2.3. Performance Analysis at Multiple Stand-Alone Stations

**Figure 8:**
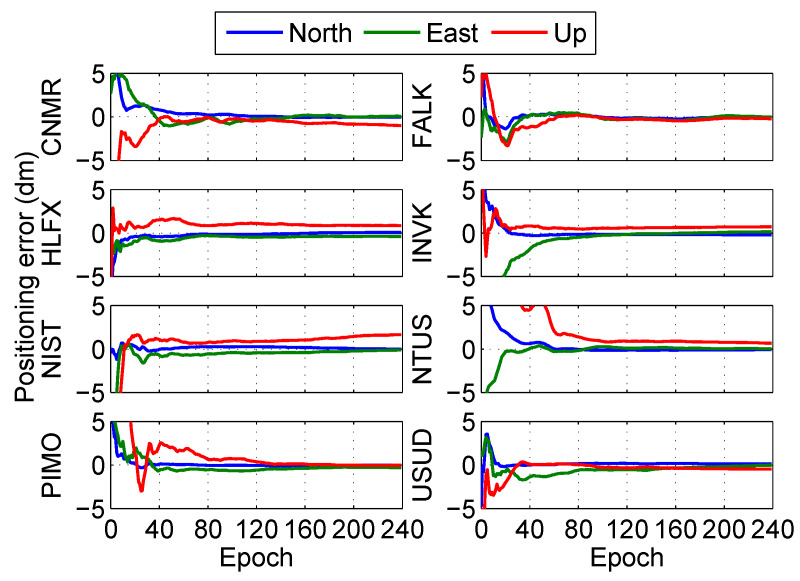
Positioning error of float solution at different stations in the post-processing PPP.

**Figure 9:**
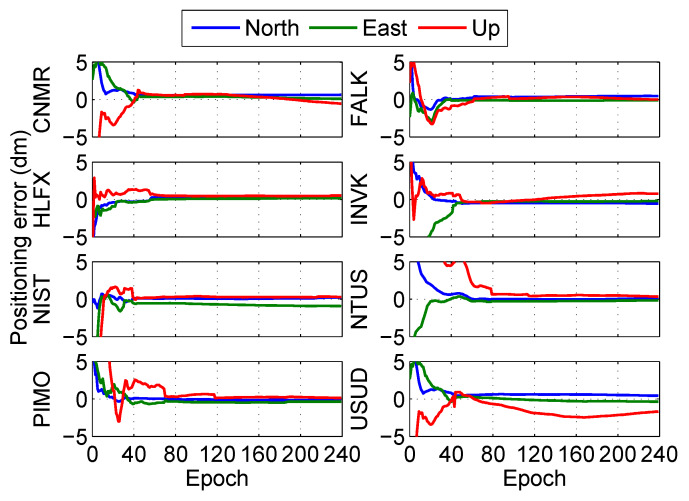
Positioning error of fixed solution at different stations in the post-processing PPP.

The positioning error at the eight stations in the post-processing PPP is shown in Figure 8 and Figure 9. The results show that the fixed solutions have better performance than float solutions at the eight stations in the post-processing PPP.

Meanwhile, the positioning performance of the eight stations is calculated in detail as shown in Table 3, where the convergence time is the instant that the positioning error in each direction is less than 0.1 m and this state can last for 20 epochs after the instant. The accuracy is the average value of the positioning error after convergence, and the precision is the root mean square of the positioning error after convergence which reflects the jitter of the positioning error. The horizontal positioning component is more accurate than the vertical component, similarly to the study by Choy et al. [40]. Compared with float solutions, the average convergence time of fixed solutions was reduced by 5.1 min (15.8%), but both the accuracy and the precision remained at the same level.

**Figure 10:**
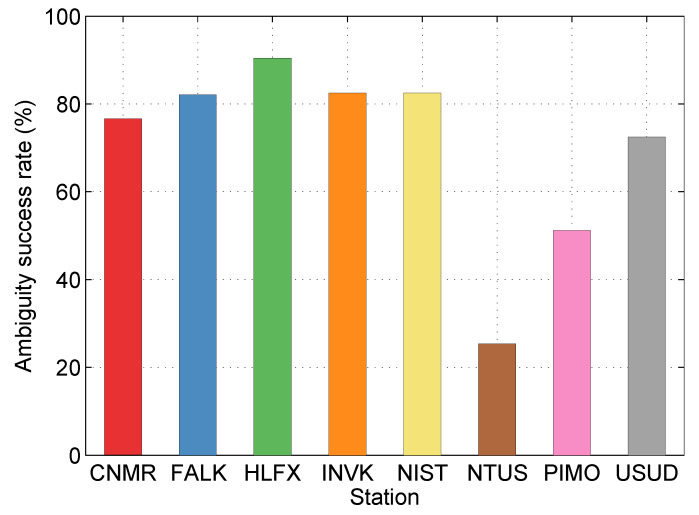
The ambiguity success rates at different stations in the post-processing PPP.

In post-processing PPP, the ambiguity success rates at different stations are shown in Figure 10. Considering that there is no estimated FCB in the first five minutes (10 epochs), the true ambiguity success rate will be higher, about 1.05 times of the statistics.

#### 5.2.4. Ambiguity Analysis

**Figure 11:**
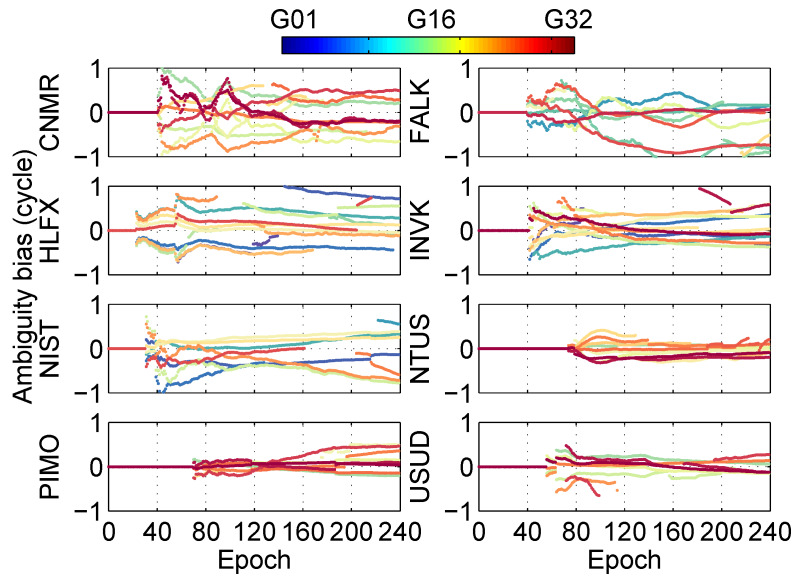
Ambiguity bias at different stations in the post-processing PPP.

In the positioning solution of ionosphere-free combination at different stations, the residual between fixed ambiguities and floating ambiguities is shown in Figure 11. The result shows that the ambiguities residuals are all within a cycle.

### 5.3. Real-Time PPP Experiment

#### 5.3.1. Stations Selection

**Figure 12:**
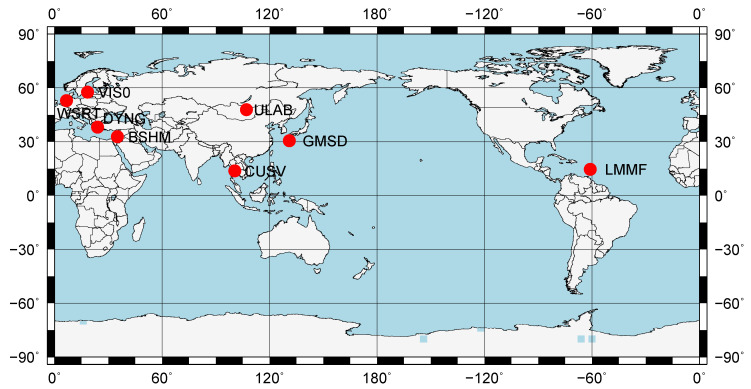
Station distribution in the real-time PPP.

In the real-time situation, according to the two constraints which are the same as the constraints in post-processing PPP, we chose the other stations around Eurasia (BSHM, CUSV, DYNG, GMSD, LMMF, ULAB, VIS0, and WSRT) as shown in Figure 12 to increase the diversity of the stations.

#### 5.3.2. Determination of the Threshold

**Figure 13:**
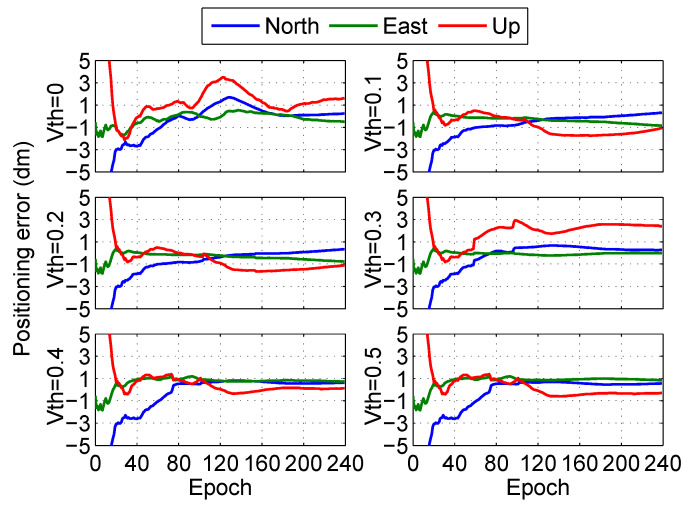
Positioning error of fixed solution in different thresholds at station BSHM in the real-time PPP.

Taking the BSHM station as an example, the influences of different thresholds $V_{th}$ in PPP-PARC are analyzed as shown in Figure 13, where $V_{th}=0$ is also the float solution.

**Figure 14:**
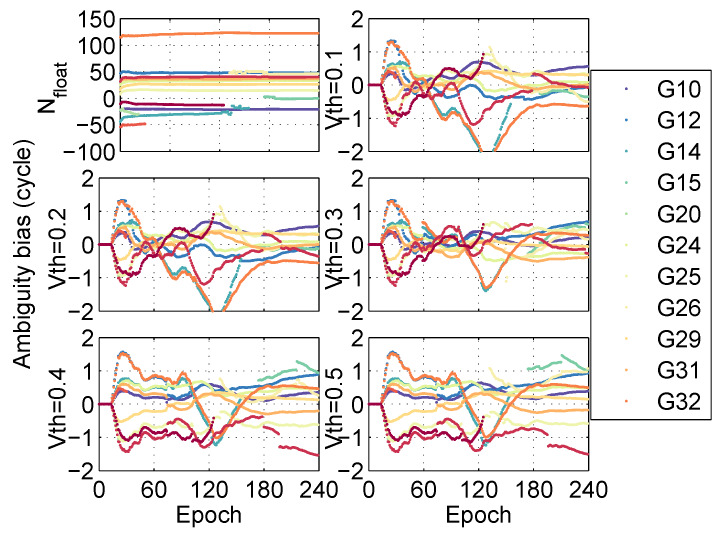
Ambiguity bias in different thresholds at station BSHM in the real-time PPP.

At the same time, the residuals between fixed ambiguities and floating ambiguities on different thresholds are analyzed as shown in Figure 14. It is found that when $V_{th}=0.3$, the performance of fixed ambiguities is the best. However, it is inconsistent with the positioning result. We can choose the threshold from others. When $V_{th}=0.1$ or $V_{th}=0.2$, most of the ambiguity residuals are closer to zero than those in the case of $V_{th}=0.4$ or $V_{th}=0.5$. According to the positioning performance of float/fixed solution in the real-time PPP and the threshold $V_{th}=0.2$ in the post-processing PPP, the threshold $V_{th}=0.2$ was also set in the real-time PPP. Subsequently, the positioning performance of the algorithm in the real-time PPP is analyzed as follows.

#### 5.3.3. Performance Analysis at Multiple Stand-Alone Stations

**Figure 15:**
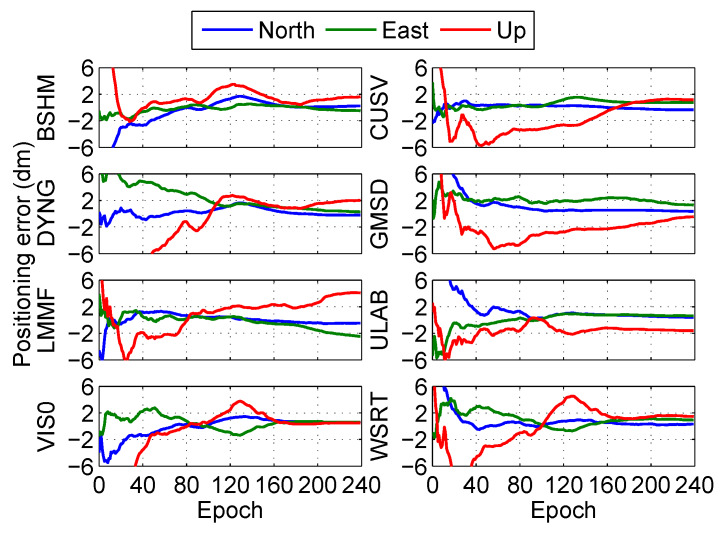
Float positioning error in different stations in the real-time PPP.

**Figure 16:**
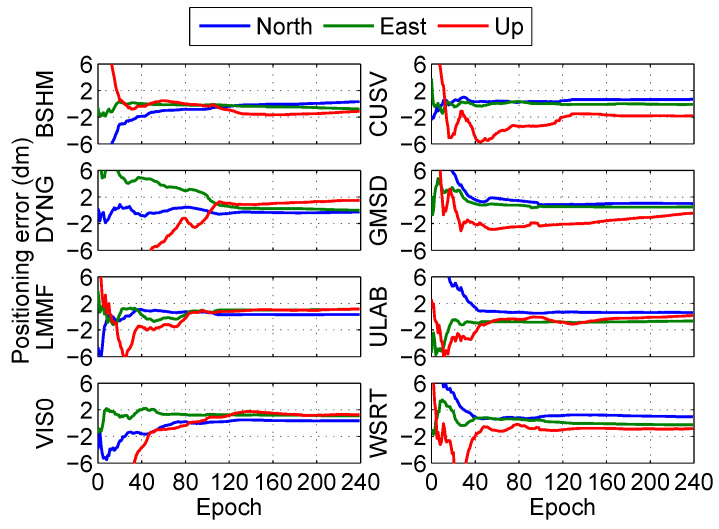
Positioning error of fixed solution at different stations in the real-time PPP.

The positioning errors of float solution at the eight stations (BSHM, CUSV, DYNG, GMSD, LMMF, ULAB, VIS0, and WSRT) under the real-time PPP are shown in Figure 15. Meanwhile, the positioning errors of the fixed solution at the eight stations under the real-time PPP are illustrated in Figure 16. The results show that the fixed solutions also have better performance than float solutions at the eight stations in the real-time PPP.

Subsequently, the positioning performance at the eight stations under the real-time PPP is calculated as shown in Table 4, where the convergence time is the instant that the positioning error in each direction is less than 0.2 m and this state can last for 20 epochs after the instant. The results show that the average convergence time of fixed solutions is reduced by 13.1 min (26.4%), compared with float solutions. Meanwhile, both accuracy and precision also remain at the same level. The convergence time is especially long at CUSV and GMSD. We analyzed the positioning performance in the next day, and we found the reason is bad observation data, which may be led by bad weather.

**Figure 17:**
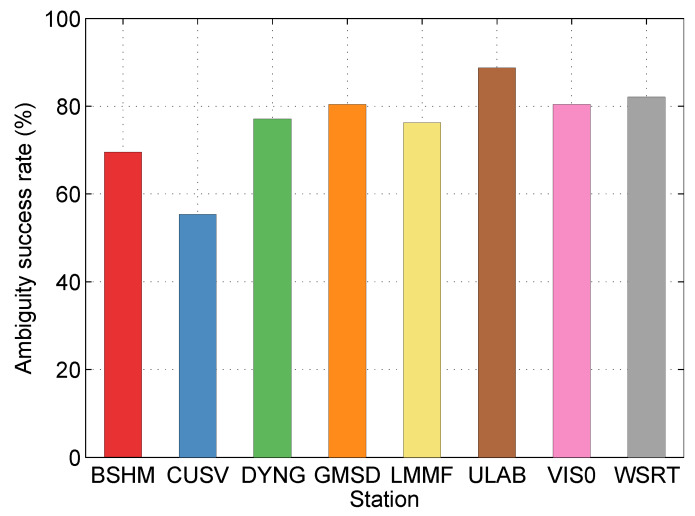
The ambiguity success rates at different stations in the real-time PPP.

In the real-time PPP, the ambiguity success rates at different stations are calculated, and they are illustrated in Figure 17. Considering that there is also no estimated FCB in the first five minutes (10 epochs), the true ambiguity success rate will be higher, about 1.05 times of the statistics.

#### 5.3.4. Ambiguity Analysis

**Figure 18:**
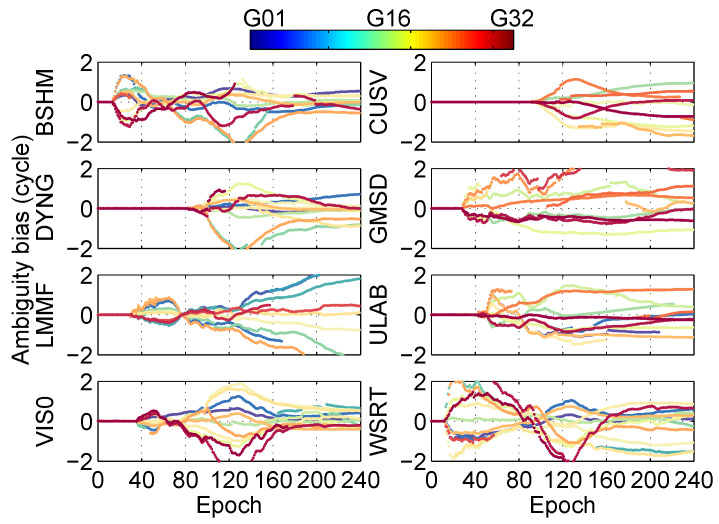
Ambiguity bias at different stations in the real-time PPP.

In the real-time PPP solution using ionosphere-free combination at different stations, the residuals between fixed ambiguities and floating ambiguities are shown in Figure 18. The result shows that the ambiguity residuals at eight stations are mostly within two cycles.

### 5.4. Extensibility Analysis

To verify the extensibility of the ambiguity residual constraint-based PPP-PAR method (PPP-PARC), the two constraints are abolished. We chose 6 globally distributed GPS stations (BJFS, BZRG, CLRT, EIL4, MAG0, OAK1) which have a stable float solution. The performances of the float solution and PPP-PARC method in post-processing and real-time PPP are shown in Table 5.

The results show that the ambiguity residual constraint-based PPP-PAR method (PPP-PARC) mainly has the same performance as the float solution. The convergence time of the PPP-PARC is three minutes longer than that of the float solution in real-time PPP at station CLRT. The convergence time of the PPP-PARC method is 9.5 min less than that of the float solution in real-time PPP at station OAK1. Therefore, the PPP-PARC method has similar performance as the float solution, when the float solution is stable in the GPS stations.

## 6. Conclusions

Ambiguity resolution is a critical prerequisite for positioning solutions. However, some of the ambiguities may have biases in the case of FCB estimation with a stand-alone receiver, PPP with partial ambiguity resolution is needed. The research is focused on a partial ambiguity fixed solution PPP without the assistance of the real-time network corrections.

Firstly, an ambiguity-fixing method for FCB estimated by a stand-alone receiver was proposed in both post-processing PPP and real-time PPP. Considering the fast convergence, the update period was changed from 15 min to 5 min. Then, the influencing factors of the ambiguity success rate in a fixed-solution were analyzed. It found that ambiguity residuals are related with the ambiguity success rates. The smaller the floating ambiguity residual is, the better the performance of ambiguity fixing is. In the case of the FCB estimation in a stand-alone receiver, the combined floating ambiguity residuals were added as the constraints to guarantee the partial ambiguity-fixing.

Subsequently, the controlled variable experiments were used to determine the ambiguity residual threshold in both post-processing PPP and real-time PPP. The result shows that the ambiguity residual threshold is 0.2 cycles. Finally, the performance of the algorithm was analyzed by independent experiments with 22 stations in both post-processing PPP and real-time positioning.

When the float solution is not good at the GPS stations, the average convergence time of fixed solutions is reduced by 15.8% and 26.4% in post-processing and real-time positioning, respectively. However, if the float solution is stable at the GPS stations, the PPP-PARC method has similar performance as the float solution.

The ambiguity residual constraint-based PPP-PAR method has room for optimization. In the future, we will continue analyzing the PPP-PARC method in detial and optimize the performance.

## Figures and Tables

**Table 1:** Error corrections.

Error Corrections	Setting
Differential Code Biases(DCB)	CODE
Cycle slip	M-W and Ionosphere Residuals
Clock slip	M-W detection and integer repair [37]
Stochastic models	Sine function model [38]
Earth rotation	Sagnac effect
Relativistic effects	General relativistic function [34]
Troposphere	Random walk + Hopfield + GMF
Antenna phase center offsets	PCV + PCO
Phase windup	Windup compensation function [39]
Earth tides correction	Solid/Pole tide

**Table 2:** General parameter settings.

Parameter	Setting
Rinex file	xxxx1000.18o
Precise orbit product	igs19962.sp3\ igu19962.sp3
Precise clock product	igs19962.clk_30s
Pole shift/ut1-utc	igs19967.erp
Antenna phase center	igs14.atx
Positioning mode	Post-processing/real-time static
Estimation algorithm	Standard Kalman Filter
Observation models	dual-frequency ionosphere-free combination
Reference coordinate	igs18P1996_all.ssc
Sampling interval	30 s
Elevation cutoff angle	$10^{\circ }$

**Table 3:** Positioning performance at different stations in the post-processing PPP.

Station	Method	CT (min)	Accuracy (mm)			Precision (mm)		
			N	E	U	N	E	U
CNMR	Float	26.5	1.7	−5.1	−73	16.9	35.9	63.4
	Fixed	23.5	59.7	27.4	23.9	59.3	30.6	52
FALK	Float	24.5	−3.5	−8.5	−23.5	16.2	26.6	29.5
	Fixed	20.5	40.2	−12.4	17	38	12.4	30.9
HLFX	Float	35	−3.1	−40.6	91.3	9.5	40.9	93.5
	Fixed	28	22.5	11.2	46	23.3	11.1	47.1
INVK	Float	26.5	−19.6	−2.5	63.2	18.9	31.8	60.8
	Fixed	21.5	−50.6	−27.8	35.4	49.9	30.5	48.7
NIST	Float	28.5	10.2	−30.3	124.8	18.8	42.2	113.3
	Fixed	20	8.9	−80.1	28.6	9.5	75.8	28.3
NTUS	Float	47	−11.9	8.6	79.3	12.3	12.4	82.5
	Fixed	40	0.1	−23.4	46.9	2.4	25	50.3
PIMO	Float	38	4.9	−35.6	7.1	5.6	44.5	36
	Fixed	35.5	−14.6	−41.7	24.1	13.7	42	32.3
USUD	Float	32.5	15	−35.2	−33.3	15.2	46.4	34.9
	Fixed	28.5	34	−24.7	−6.8	33.1	30.3	9.8
Average	Float	32.3	−2	−18.7	29.5	14.2	35.1	64.2
	Fixed	27.2	12.5	−21.4	26.9	28.7	32.2	37.4

**Table 4:** Positioning performance at different stations in the real-time PPP.

Station	Method	CT (min)	Accuracy (mm)			Precision (mm)		
			N	E	U	N	E	U
BSHM	Float	24.5	31.9	−4.4	147.8	80	30.5	173.9
	fixed	18.5	−21.9	−43.1	−116.3	68.3	41.2	113.4
CUSV	Float	71.5	−19.2	82.2	64.1	23.7	86.5	105
	fixed	60.5	64.5	−5.9	−173.5	64.3	6.8	173.3
DYNG	Float	71	7.7	67	148.8	42.8	79.9	150
	fixed	51	−34.5	27.4	115.4	35.9	44.9	114.7
GMSD	Float	98.5	42.6	155.7	−86.4	43.2	159.2	96.1
	fixed	67.5	100.5	49.1	−111.5	100	49.3	130.6
LMMF	Float	37	−19	−84.4	245.3	41	115.7	242.6
	fixed	26.5	32.6	94.1	92.5	46.3	92.4	100.8
ULAB	Float	30	68.5	64.5	−138.8	81.6	64.9	144.2
	fixed	21.5	64.7	−76.3	−32.7	66.3	77.8	56
VIS0	Float	28.5	66.4	32.6	89.6	76.8	77.6	143.8
	fixed	26.5	32.8	117.5	115.8	36.7	122.3	119.7
WSRT	Float	36.5	66.4	67.7	165.6	50.3	85.7	212
	fixed	20.5	107.6	−9.7	−86.9	101.3	37.3	89.4
Average	Float	49.7	27.4	47.6	79.5	54.9	87.5	158.4
	fixed	36.6	43.3	19.1	−24.7	64.9	59	112.2

**Table 5:** Positioning performance at different stations with a stable float solution.

Station	Positioning Type	Method	CT (min)	Accuracy (mm)			Precision (mm)		
				N	E	U	N	E	U
BJFS, China	Post-processing	Float	17.5	−23.2	−47.1	50.0	30.6	52.8	56.7
		fixed	17.5	−2.5	32.8	−68.9	8.9	33.0	70.0
	Real-time	Float	20.5	31.7	−4.9	119.0	69.7	46.8	116.7
		fixed	20.5	135.3	182.0	−79.6	135.5	164.8	98.8
BZRG, Italy	Post-processing	Float	21.5	24.4	−11.9	−38.6	22.9	32.7	41.6
		fixed	21.5	12.7	−35.9	−30.1	14.4	39.0	42.2
	Real-time	Float	51	63.1	35.2	13.7	97.9	65.1	600.9
		fixed	51	141.1	84.4	87.9	132.4	84.2	604.6
CLRT, Canada	Post-processing	Float	8.5	−25.3	−24.5	37.0	30.2	24.5	41.6
		fixed	8.5	−6.0	−63.8	102.5	16.4	62.1	103.8
	Real-time	Float	23	−31.4	−29.4	48.2	66.5	75.8	492.5
		fixed	27	63.3	−58.8	204.4	69.3	73.6	523.2
EIL4, USA	Post-processing	Float	6	−6.8	21.3	60.9	18.5	35.5	54.3
		fixed	6	−40.5	116.1	15.4	38.5	109.9	26.9
	Real-time	Float	40	−55.9	76.2	28.4	70.1	85.4	191.4
		fixed	40	−83.7	80.2	44.7	81.7	86.9	190.6
MAG0, Russia	Post-processing	Float	26	16.7	−29.8	−40.1	15.7	40.0	42.7
		fixed	26	18.4	−57.3	55.9	17.6	57.4	62.9
	Real-time	Float	38.5	49.6	68.2	−199.0	57.5	67.9	261.4
		fixed	38.5	41.2	119.1	40.5	51.2	96.1	141.4
OAK1, UK	Post-processing	Float	7	25.0	−24.5	−35.2	24.2	33.3	34.1
		fixed	7	−25.7	−23.1	58.7	25.1	31.4	55.8
	Real-time	Float	26	63.0	45.7	180.2	62.4	59.2	256.9
		fixed	16.5	−97.6	65.8	263.8	90.0	78.9	242.5

## Abbreviations

The following abbreviations are used in this manuscript:

CORS	continuously operating reference station
DSC	decoupled satellite clock
DCB	differential code bias
FCB	fractional cycle bias
GMF	Global Mapping Function
GNSS	Global Navigation Satellite System
GPS	Global Positioning System
GPST	Global Positioning System Time
IRC	integer recovery clock
M-W	Melbourne-Wubbena detecting
PCO	Antenna Phase Center Offsets
PCV	Antenna Phase Center Variations
PPP	precise point positioning
PPP-AR	PPP with ambiguity resolution
PPP-PAR	PPP with partial ambiguity resolution
PPP-PARC	ambiguity residual constraint-based PPP with partial ambiguity resolution
PPP-RTK	PPP with real-time kinematic
SA	selective availability
UHD	uncalibrated Hardware Delay
UPD	uncalibrated phase delay
UTC	Universal Time Coordinated
